# Multiple Independent Introductions of HIV-1 CRF01_AE Identified in China: What Are the Implications for Prevention?

**DOI:** 10.1371/journal.pone.0080487

**Published:** 2013-11-25

**Authors:** Yassir F. Abubakar, Zhefeng Meng, Xiaoyan Zhang, Jianqing Xu

**Affiliations:** 1 Shanghai Public Health Clinical Center and Institutes of Biomedical Sciences, Key Laboratory of Medical Molecular Virology of Ministry of Education/Health at Shanghai Medical College, Fudan University, Shanghai, China; 2 State Key Laboratory of Infectious Disease Control and Prevention (CDC), Beijing, China; The University of Hong Kong, China

## Abstract

**Background:**

HIV-1 CRF01_AE accounts for an important fraction of HIV infections in Asia including China, but little is known about the phylogenetic and evolutionary history of this CRF (circulating recombinant form). In the current study, we collected a large number of 1,957 CRF01_AE gag p17 sequences with known sampling year (1990-2010) from 5 global regions representing 15 countries to better understand the phylogenetic relationships and epidemic history of CRF01_AE strains in China.

**Methodology/Principal Findings:**

CRF01_AE gag p17 sequences were retrieved from public databases to explore phylogenetic relationships and phylogeographic dynamics of CRF01_AE in Asia by using maximum-likelihood phylogenetics and Bayesian coalescent-based analyses. We found close phylogenetic relationships between sequences from Thailand, Vietnam and China. Moreover, at least 5 independent introductions and 5 independent autochthonous clades of CRF01_AE, which descended from Thailand or Vietnam were identified in China from 1991 through 2003.

**Conclusion/Significance:**

The current study not only defines the migration of CRF01_AE clades to/in Asia, but also demonstrates the criticalness of identifying the circulating strains in the population for the development of vaccine and microbicides.

## Introduction

Human immunodeficiency virus type 1 (HIV-1) sequences belonging to the pandemic group M are classified into nine subtypes (A–D, F–H, J, and K), six sub-subtypes (A1-A4, and F1- F2), and a variety of inter-subtype recombinant forms (Los Alamos HIV sequence database: http://hiv-web.lanl.gov/). CRF01_AE as one of HIV-1 circulating recombinant forms (CRF) is considered globally the fifth largest subtype and found in South and South-East Asia, East Asia and a small number in Central Africa [1]. Consequently, CRF01_AE had been reported as prevalent and responsible for the vast majority of infections in South and South East Asia[1,2]. In China, as part of East Asian region, CRF01_AE represents one of major HIV-1 genotypes reportedly associated with particular risk populations[1,3,4].

Numerous studies reported that CRF01_AE had led a new epidemic in many major HIV-1 prevalent provinces and municipalities of China, and displayed a complex characteristics in these locations [5,6]. Moreover, CRF01_AE, which was the dominant strain in the sexual-risk population [6], apparently played more important role in MSM (men having sex with men) population [3]. Additionally, the increasing percentages of CRF01_AE infections in many Chinese cities exceeded those of the previously predominant subtype B [3,7,8].

So far, little is known about the phylogenetic relationships and epidemic history of CRF01_AE clades in Asia at large and China in particular. Furthermore, most studies in China had been conducted at provincial level [3,9-11]; therefore, much is needed to explore CRF01_AE epidemic history by involving all Asian clades to obtain more comprehensive picture of CRF01_AE dynamics.

By using a combination of phylogenetic analyses and Bayesian coalescent-based approach in this study, we analyzed a total of 1,957 HIV-1 CRF01_AE *gag* p17 sequences from 5 global regions containing 15 countries including China (Table 1), to better understand the phylogenetic relationship between global and Asian clades, and to explore in greater detail the epidemic history of CRF01_AE in China. 

**Table 1:** Region, country, number and sampling date of all sequences used in the study.

**Region**	**Country (n)**	**N**	**Sampling date**
Central Africa **(CF**)	Central African Rep. (CAR)	**3**	1990
	Democratic Republic of Congo (CD)	**9**	1984
West Africa (WA)	Cameroon (CM)	**4**	2007
South and South-East Asia (**SSE**)	Afghanistan (AF)	**1**	2007
	Indonesia (ID)	**8**	1993-2007
	Iran (IR)	**1**	2010
	Myanmar (MM)	**10**	1997-1999
	Singapore (SG)	**28**	1996
	Thailand (TH)	**1474**	1990-2009
	Vietnam (VN)	**218**	1997-2008
East Asia (EA)	China (CN)	**182**	1996-2010
	Japan (JP)	**9**	1993-2000
West and Central Europe (WCE)	Denmark (DK)	**2**	1996-2000
	UK (GB)	**1**	2005
	Sweden (SE)	**7**	2006-2010
**Total**	15	**1,957**	**1990-2010**

Our results demonstrated the close phylogenetic relationship between Thailand, Vietnam and Chinese CRF01_AE sequences. Interestingly, we found multiple independent introductions and multiple independent autochthonous clades of HIV-1 CRF01_AE circulating in different geographic locations of China, which probably descended from Thailand, Vietnam or both. Yunnan province of China was identified as the earliest introduction site, and together with Guangxi, Fujian and Liaoning were demonstrated as locations harboring those clades. Those clades, however, were indicated as independently major or sporadic introductions, which likey took place from 1991 through 2003.7.

## Materials and Methods

### Sequence dataset and Alignment

All HIV-1 CRF01_AE *gag* p17 sequences matching the selected genomic region (nt 913–1111 relative to HXB2 clone) were retrieved from Los Alamos HIV Database (http://hiv.lanl.gov). Of note, HIV-1 gag *p17* gene had been used previously for HIV-1 genotyping or phylogenetic analysis and appeared less prone to recombination [2,6,12-14]. Additionally, sequences of this HIV-1 genomic region cover uniquely most of CRF01_AE prevalent Asian countries and key provinces in China as well (http://hiv.lanl.gov); a feature qualifies this genomic region to fit the study of CRF01_AE epidemic history in China. And, as a preliminary step to test the high-quality and eligibility of this genomic region, all gag p17 sequences were subjected to bioinformatic tools that available at Los Alamos database website.

Countries were regrouped into geographic regions according to the classification proposed by Hemelaar et al [1]. To improve the accuracy of phylogenetic inference, subtype assignment of all sequences was confirmed by the REGA HIV subtyping tool v.2[15]. All sequences with evidence of erroneous subtype assignment or evidence of inter-subtype recombination were excluded. The final dataset was composed of 1,957 CRF01_AE *p17* sequences sampled from blood and collected between 1990-2010, which represented 15 countries (Table 1). Sequences were obtained through the utilization of PBMCs, serum or plasma samples of HIV-1 positive individuals, and methods of extraction and amplification were deliberately detailed in their relative studies [6,8,16-20]. All sequences were aligned with HIV-1 subtype reference sequences (http://www.hiv.lanl.gov sequence alignment page) using GeneCutter (http://www.hiv.lanl.gov GeneCutter page). Alignment quality was checked manually in Bio-Edit V7.8 [21] to ensure that the alignments did not contain obvious problems and that they were correctly codon aligned.

### Phylogenetic analysis of HIV-1 CRF01_AE sequences

Maximum-likelihood (ML) phylogenetic tree was inferred under the GTR+I+C4 nucleotide substitution model (default model) after reconstruction with PhyML program [22] using an online web server [22]. Heuristic tree search was performed using the SPR branch-swapping algorithm and the reliability of the obtained topology was estimated with the approximate likelihood-ratio test (αLRT) [23] based on the Shimodaira-Hasegawa-like (SH-like) procedure. The ML trees were visualized using the FigTree v1.3.1 program [24]. Only lineages in ML tree supported by approximate likelihood ratio test (aLRT) ≥0.95 were selected. We assessed the temporal signal of p17 dataset as previously described in ML tree construction and clock-like behavior of the resultant data was also evaluated through regressing root-to-tip distance by using Path-O-Gen v1.3[25,26]. This analysis effectively provides a measure of the amount of variation in genetic distances explained by sampling time.

### Phylogeographic Analysis of HIV-1 CRF01_AE lineages

The evolutionary rate (µ, units are nucleotide substitutions per site per year, subst./site/year), the age of the most recent common ancestor (tMRCA), and the probable geographic origin of Chinese CRF01_AE lineages were estimated using the Bayesian Markov Chain Monte Carlo (MCMC) approach implemented in BEAST software package v1.6.2 [27,28]. The best nucleotide substitution model for all datasets was evaluated by Mega software v5.2. MCMC analyses were performed using the Hasegawa-Kishino-Yano (HKY) nucleotide substitution model [29,30] with a gamma-distributed model among site rate variation using four rate categories (C4) [29,31], an uncorrelated Lognormal relaxed molecular clock model [32], and Bayesian Skyline coalescent tree prior [33]. Two separate MCMC chains were run for 8x10^8^ generations and adequate chain mixing was checked by calculating the effective sample size (ESS) after excluding an initial 10% for each run using program TRACER v1.4 [34]. MCMC runs converged to almost identical values and combined estimates showed ESS values >150. Maximum clade credibility (MCC) trees were summarized from the posterior distribution of trees with TreeAnnotator and visualized with FigTree v1.3.1. For the exclusion that substitution rate and tMRCA estimates could be biased by either over- or under-representation of countries under study, BEAST analysis was applied to a dataset of randomly selected p17 sequences containing equal number of sequences (i.e. 15 each) per country in case of countries with abundant sequences, plus 3 sequences from less represented countries. The randomization process was repeated several times and nucleotide substitution rates and tMRCAs were estimated every time [26]. Furthermore, to confirm the latter outcome, the full gag (HXB2 numbering: 790-2235) dataset of the same randomly selected dataset was again compared with both gag p17 datasets. Once again no significant difference between parameters was detected (Data not shown).

## Results

### Sequences collected from database and used for the analyses

Among all HIV-1 CRF01_AE *gag p17* gene sequences that were downloaded, we first discarded all sequences without geographical information or identified as redundant sequences. Sequences which were not identified as CRF01_AE (i.e. intersubtype recombinants) by the REGA-subtyping tool were removed as well; and we kept only one sequence per patient. Finally, the utilized dataset included a total of 1,957 CRF01_AE gag p17 (nt 913–1111 relative to HXB2 clone), covering all five geographic regions included in the study (West Africa, Central Africa, West and Central Europe, East Asia, and South and Southeast Asia) (Table 1). This dataset was employed to characterize the phylogenetic relationships between CRF01_AE sequences of all involved regions, and to delineate with more confidence the relationships within Asian sequences, which was the main focus of the study.

### Phylogenetic relationships between HIV-1 CRF01_AE sequences

**Figure 1:**
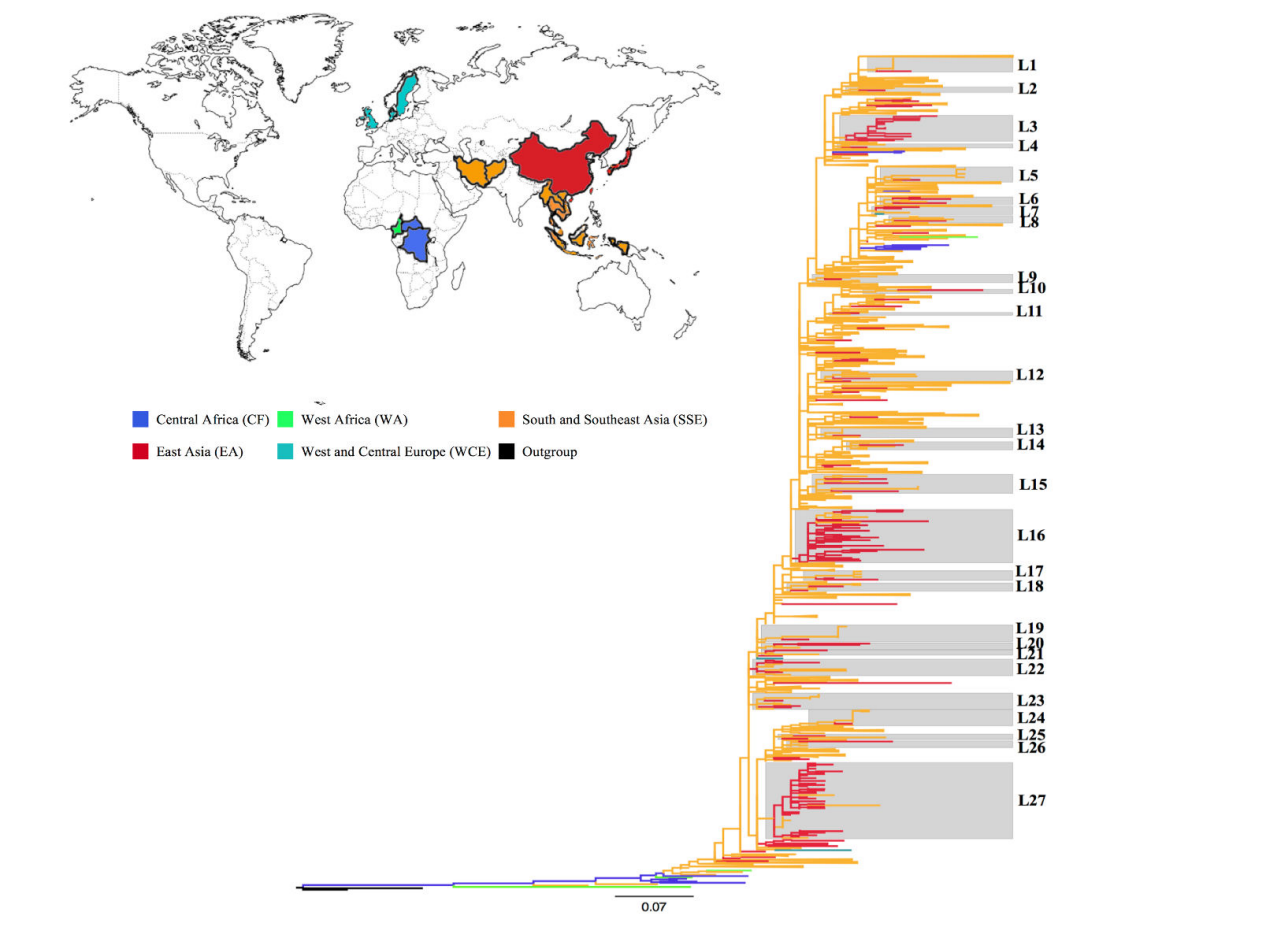
ML tree of HIV-1 CRF01_AE gag p17 sequences from 5 global regions (15 countries). The boxes highlight the position of the Chinese CRF01_AE lineages. The color of the branches represents the geographic region from where the CRF01_AE sequences originated, according to the map given at the top left of the figure. For visual clarity, some clades comprised of sequences sampled from SSE region were collapsed in orange triangles. The tree was rooted using outgroup (black branches). Horizontal branch lengths are drawn to scale with the bar at the bottom indicating nucleotide substitutions per site.

Maximum likelihood tree was reconstructed using a total of 1,957 CRF01_AE *gag* p17 sequences from all 5 global regions (Figure 1). Figure 1 demonstrates the maximum likelihood (PhyML) tree, indicating that all CRF01_AE strains were originally from Central Africa and sequences from South and Southeast Asia formed the earliest Asian subcluster. Of note, all sequences of the latter subcluster were Thai sequneces. Thus, Thailand represented the origin of all Asian CRF01_AE strains. The highlighted lineages in grey represent 27 lineages (L1-L27) that highly supported by aLRT value ≥0.95 at the first or second node [35]. Interestingly, all lineages (n=27) contained interspersed Asian sequences mainly from South and Southeast Asia (SSE) region and from East Asia, indicating close phylogenetic relationships between these two regions. However, only one lineage (i.e. L7) contained in addition to the above-mentioned regions one sequence from West and Central Europe region (i.e. GB). Of more interest, 13 lineages composed only of one Chinese sequence, while the remaining 14 lineages contained ≥2 Chinese sequences (designated major Chinese lineages).

A closer view of those 14 Asian lineages showed that Chinese sequences branched with sequences from Thailand, Vietnam or both, demonstrating close phylogenetic relationships between those CRF01_AE sequences. Our findings are in agreement with previous studies indicating HIV-1 CRF01_AE transmission involving the 3 countries [6,17]. Moreover, major Chinese clades phylogenetically related to Thai sequences found in lineages L16, L17, L22, L23 and L27, while those related to Vietnam contained in lineages 6 and 20 (Figure S1). 

Furthermore, those Asian lineages contained 5 major Chinese subclusters in which the latter branched with Thai sequences and represented sequences from Guangxi (southern Chinese province bordering Vietnam) (L16, L17), Hubei (central province) (L22) and 2 clades from Liaoning (northeastern Chinese province) (L27). Whereas, another 2 Chinese subclusters branched with Vietnam sequences represented Fujian (southeastern province) (L6) and Guangxi (L20) sequences. 

Our results pointed directly to close phylogenetic relationships between CRF01_AE clades from Thailand or Vietnam, and sequences of the above mentioned geographic locations inside China (Figure S1), an outcome that is consistent with previous epidemiologic reports [36,37].

### Origin of HIV-1 CRF01_AE sequences circulating in China

 To determine with more confidence the most probable origin of those Chinese CRF01_AE lineages, we applied a Bayesian statistical framework that allows ancestral reconstruction of the locations at the interior nodes of Bayesian tree while accommodating phylogenetic uncertainty. We combined Chinese CRF01_AE sequences and those from countries previously pointed out by ML tree as being the most probable ancestral locations of Chinese lineages (Central African Republic, Thailand and Vietnam) to perform this analysis. For Chinese sequences, we included only sequences of China’s peripheral provinces, wherein earliest CRF01_AE infections in China were identified and probably constituted the entry sites. The selected geographic locations included Yunnan (n=6), Guangxi (n=47), Fujian (n=8) and Liaoning (n=47). Global sequences were those that branched with one or more Chinese sequences until the second ancestral node in the ML phylogenetic tree, which included Thailand (n=147), Vietnam (n=20) and CAR (n=3). Sequences from other countries were excluded due to poor representation (≤2 sequences). Ultimately, a total of 278 sequences were used for the reconstruction of Bayesian maximum clade credibility (MCC) tree.

**Figure 2:**
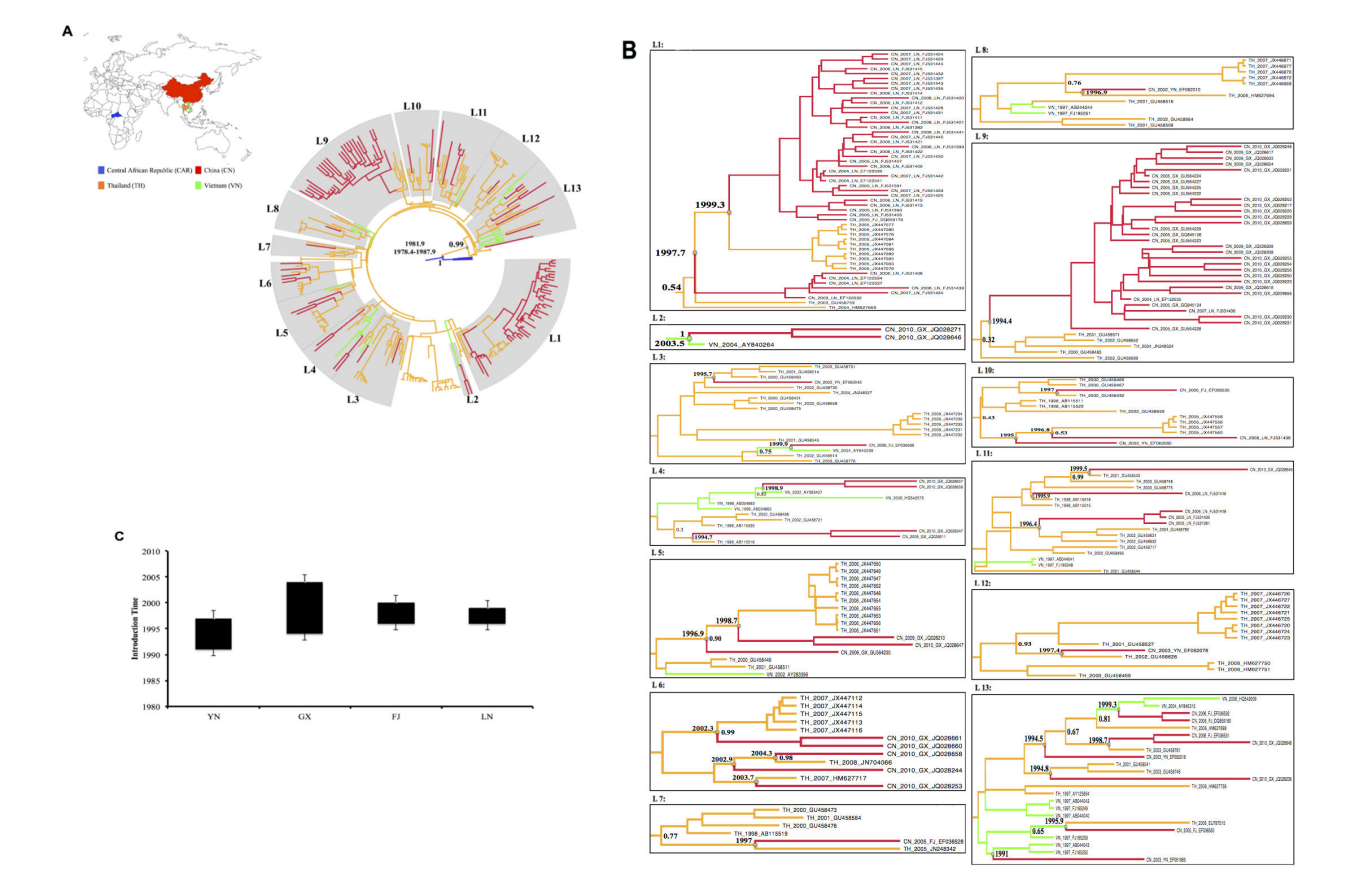
Time-scaled Bayesian Maximum Clade Credibility (MCC) tree. **A**: Overall topology of MCC tree of the Chinese CRF01_AE lineages and the most closely related global sequences. Branches are colored according to the most probable location state of their descendent nodes. The color of the branches represents the geographic region from where the CRF01_AE sequences originated, according to the map given at the top left of the figure. The boxes highlight the position of the Chinese CRF01_AE clades. The first (CF) and second (TH) nodes representing initial nodes lineages to all lineages together with posterior probability values 1 and 0.99, respectively, are indicated. The median and 95% HPD interval of the tMRCA at the root of the tree is indicated. The tree was automatically rooted under the assumption of a relaxed molecular clock. **B**: Close view of the Chinese CRF01_AE clades and the most closely related Asian sequences in MCC tree. The color of branches represents the geographic region from where the sequence originated. The names of CRF01_AE strains include reference to country origin, year of isolation, province (Chinese provinces) and GenBank Accession number. Countries represented are Central African Republic (CF), Thailand (TH), Vietnam (VN) and China (CN), the later represented by provincial sequences: Yunnan (YN), Guangxi (GX), Fujian (FJ) and Liaoning (LN). The posterior probability (PP) support values are indicated only at key nodes. **C**: Estimated time-range of HIV-1 CRF01_AE earliest introductions into 4 border provinces (Yunnan (YN), Guangxi (GX), Fujian (FJ) and Liaoning (LN)) in China, the 95% highest posterior density (HPD) credible regions are provided as the range of state tMRCA.

Figure 2A summarizes the overall topology of the obtained Bayesian MCC tree. It included 13 Asian lineages (L1-L13) in grey color that contained Chinese sequences. Figure 2B displays a closer view of the 13 lineages; interestingly, 10 major Chinese clades (≥2 sequences) contained within 8 Asian lineages represented most likely independent CRF01_AE clades. 

 Lineage 1 contained 2 independent Chinese clades from Liaoning, which branched with Thai sequences. However, lineage 1 is poorly supported (Posterior Probability (PP) value=0.54), raising uncertainty about those 2 clades as descendants from Thailand. Another Liaoning subcluster formed within lineage 11, though branched with Thai sequences, the node was not supported (PP<0.50). 

 Lineages 2 and 4 composed of independent Chinese clades from Guangxi, which descended from Vietnam with high support at the first or second node i.e. PP = 1 and 0.85, respectively. Furthermore, another 2 Guangxi clades descended from Thailand demonstrated in lineages 5 and 6 with high support (PP value = 0.90 and 0.99, respectively). Together, our results pointed to 4 major Chinese CRF01_AE clades from Guangxi most likely introduced from Thailand (n=2) and Vietnam (n=2). Other major Guangxi clades observed in lineages 4 and 9, but without PP support (PP=0.30, 0.32, respectively), which likely resulted from internal spread network.

 In addition, lineage 13 contained another independent Chinese clade from Fujian that descended from Vietnam, and supported by a PP value equal to 0.81 at the second node.

 Altogether, ancestral reconstruction of the locations at the interior nodes of Bayesian tree revealed that the most probable place at the root of those major Chinese CRF01_AE clades could be traced to a neighboring country with high confidence including: Thailand for Guangxi clades in lineages 5 and 6; and Vietnam for Guangxi clades in lineages 2 and 4, and Fujian clade in lineage 13. The phylogenetic relationships between the 4 mentioned provinces/regions of China, Thailand and Vietnam were documented previously [3,6,37,38].

### Time-scale of CRF01_AE introductions into China

By employing the time-scale Bayesian MCC tree, we estimated the age of the MRCA (*tMRCA*) of those Chinese CRF01_AE clades in all 13 Asian lineages. As mentioned above, we considered Chinese clades with ≥2 sequences in lineages as major introductions and <2 as sporadic introductions into China. The coefficient of substitution rate variation was higher than zero (0.56; 95% HPD: 0.32–0.79), which supported the use of a relaxed molecular clock model. The rate of evolution of gag p17 gene was estimated to be 2.58× 10^-3^ substitutions per site per year. 

The tMRCA of the Asian CRF01_AE lineage was estimated at 1981.9 (95%, CI: 1978.4-1987.9), which is within the range of previous estimations [3].

The tMRCAs of major Chinese clades were estimated at: 1996.4 (95%, CI: 1994.5-1999.7) and 1997.7 (95%, CI: 1996.1-2000.7), and 1999.3 (95%, CI: 1996.2-2000) for Liaoning clades in lineage 11 and 1, respectively; 1994.4 (95%, CI: 1995.3-1997.4), 1994.7 (95%, CI: 1993.3-1997.1), 1998.7 (95%, CI: 1997.4-2004), 1998.9 (95%, CI: 1997.8-2001.6), 2002.3 (95%, CI: 2001.2-2006.1) and 2003.5 (95%, CI: 2001.5-2006) for Guangxi clades in lineages 9, 4, 5, 4, 6 and 2, respectively; 1999.3 (95%, CI: 1998.8-2003.3) for Fujian in lineage 13 (Figure 2B). The estimated introduction time of those major lineages is consistent with previous epidemiological data and reports [3,11,37,39]. 

Figure 2C summarizes the time-range of probable CRF01_AE introductions into China including all clades together (major and sporadic). Interestingly, a Chinese clade from Yunnan (southwestern province bordering Myanmar, Laos and Vietnam) in lineage 13 emerged as the earliest CRF01_AE clade in China and was dated back to 1991 (95%, CI: 1992.4-1994.2). Although this clade (one sequence) was not supported in lineage 13, the tMRCA estimation coincided with recently published study (i.e. 1990.9)[40]; even though, it should be considered with caution.

## Discussion

 In this study we analyzed HIV-1 CRF01_AE *gag p17* sequences from 5 global regions (15 countries) to better understand the phylogenetic relationships and epidemic history of this CRF in Asia and China in particular. The analysis of 1,957 globally distributed sequences most likely provided an adequate representation of global CRF01_AE diversity, and provided not only additional information on the subtype epidemic in Asia, but also in other parts of the world. Our study revealed that all CRF01_AE strains were originated in Central African Republic and then spread into South and Southeast Asian region, wherein existed an important triangle of transmission route i.e. Thailand - Vietnam - China, and Thailand - China, leading obviously to multiple independent introductions of CRF01_AE into China from neighboring countries. 

The earliest introduction probably occurred in 1991 (Lineage 13) as a sporadic event from Vietnam to Yunnan province, which seemingly not the cause of an outbreak or spread to other regions, even though this date should be considered cautiously. Guangxi represented the second earliest site to receive HIV-1 CRF01_AE in 1994.4, which resulted in an outbreak in Guangxi and formed the major cluster (Lineage 9) in MCC tree; Interestingly, it remains uncertain where the sequences were derived from since the PP value for Thailand-Guangxi subcluster was 0.32. Subsequently, Guangxi received further independent transmissions from both Vietnam in 1998.9 and 2003.5, and Thailand in 1994.7, 1998.7 and 2002.3, indicating frequent travelling between Guangxi and Thailand or Vietnam. Liaoning apparently was the third site of CRF01_AE introduction in which major introductions took place in 1996.4, 1997.7 and 1999.3. Again, the origins of those independent introductions remain uncertain because the PP value for Thailand - Liaoning was below 0.5. Perhaps, Liaoning clades in lineages 1 and 9 were derived from earliest strains of Yunnan; however, the linkages were not significantly established due to the scarcity of sequences from Yunnan (only 6 sequences were used) at the time of the study. Fujian represented the fourth site to receive CRF01_AE introduction in 1999.3 from Vietnam. These observations are in accordance with previous reports [3,6,37,41-43]. It is worth mentioning that lack of sequences from unrepresented countries bordering China, in addition to over-representation of others probably impeded the study to illustrate more phylogenetic relationships and introductions. 

As widely known, the spread of HIV-1 varients is mainly driven by means of sexual contact including both heterosexual [44] and homosexual [3], in addition to blood donation, injection drug use and mother to child transmission [45], which are known routes of transmission in Asia. CRF01_AE transmission in particular has been related remarkably to sexual contact [16,45,46]. Hence, as commercial sex is considered to be an occasional practice, it had most likely contributed to the earliest sporadic transmissions and interspersed linkages between CRF01_AE variants, an observation that indicated by our study and previous reports [6,16,41,43,46]. Nevertheless, the current study was precluded from deriving a route-linked conclusion on lineages, due to the lack or limitation of information describing patients' transmission routes in HIV database. However, as the travelling population could significantly influence the transmission patterns, multiple introductions of CRF01_AE from Vietnam and Thailand were likely driven by the increasing inter-country travels.

It remains unclear why only two large clusters were formed in spite of many independent introductions. The established transmission from neighboring countries into China with high support failed to form any large cluster (>2 sequences), indicating the unlikeliness of those strains to cause any significant outbreak. In contrast, we were unable to directly link the two large clusters of Guangxi and Liaoning to any strains in neighboring countries, suggesting that those two clusters were most likely autochthonous clades of uncertain origin, knowingly a prolonged evolution is required for those strains to exist as fully imprinted Chinese strains. In this regard, it is critical to identify the populous strains circulating among the population for the development of effective vaccine and microbicides. And certainly further surveillances to explore the biological differences between strains derived from large clusters and sporadically transmitted strains are highly needed.

In conclusion, this study demonstrates the existence of multiple independent introductions and several autochthonous transmission networks of HIV-1 CRF01_AE in China that probably resulted in more complicated pattern of CRF01_AE transmission in this most populous nation. Our analyses not only deepen the understanding on the epidemic history of CRF01_AE in Asia, but also drive the attention to the paramount importance of continuous surveillance of CRF01_AE variants in China to adapt treatment, vaccine and microbicide strategies. 

## Supporting Information

Figure S1 **Close view of ML lineages containing Chinese CRF01_AE clades and the most closely related global sequences.** The color of branches represents the geographic region from where the sequence originated, as explained in Figure 1. The names of CRF01_AE strains include reference to geographic region, country of origin, year of isolation, province (in case of Chinese sequences) and GenBank Accession number. Geographic regions represented are South and Southeast Asia (SSE), West and Central Europe (WCE), and East Asia (EA). Countries represented are Thailand (TH), Vietnam (VN), Japan (JP), Singapore (SG), Myanmar (MM), Great Britain (GB), and China (CN). Chinese provinces represented are Yunnan (YN), Guangxi (GX), Fujian (FJ), Liaoning (LN), Hebei (HEB), Hubei (HUB) and Henan (HEN). The aLRT support values are indicated only at key nodes.
(DOCX)
