# Supplementary figures and images for: Multiple Independent Introductions of HIV-1 CRF01_AE Identified in China: What Are the Implications for Prevention?
Source: PLoS One. 2013 Nov 25;8(11):e80487. doi: 10.1371/journal.pone.0080487 (PMC3839914; doi:10.1371/journal.pone.0080487)

**Supplementary Information:**

**Figure S.**

L1:


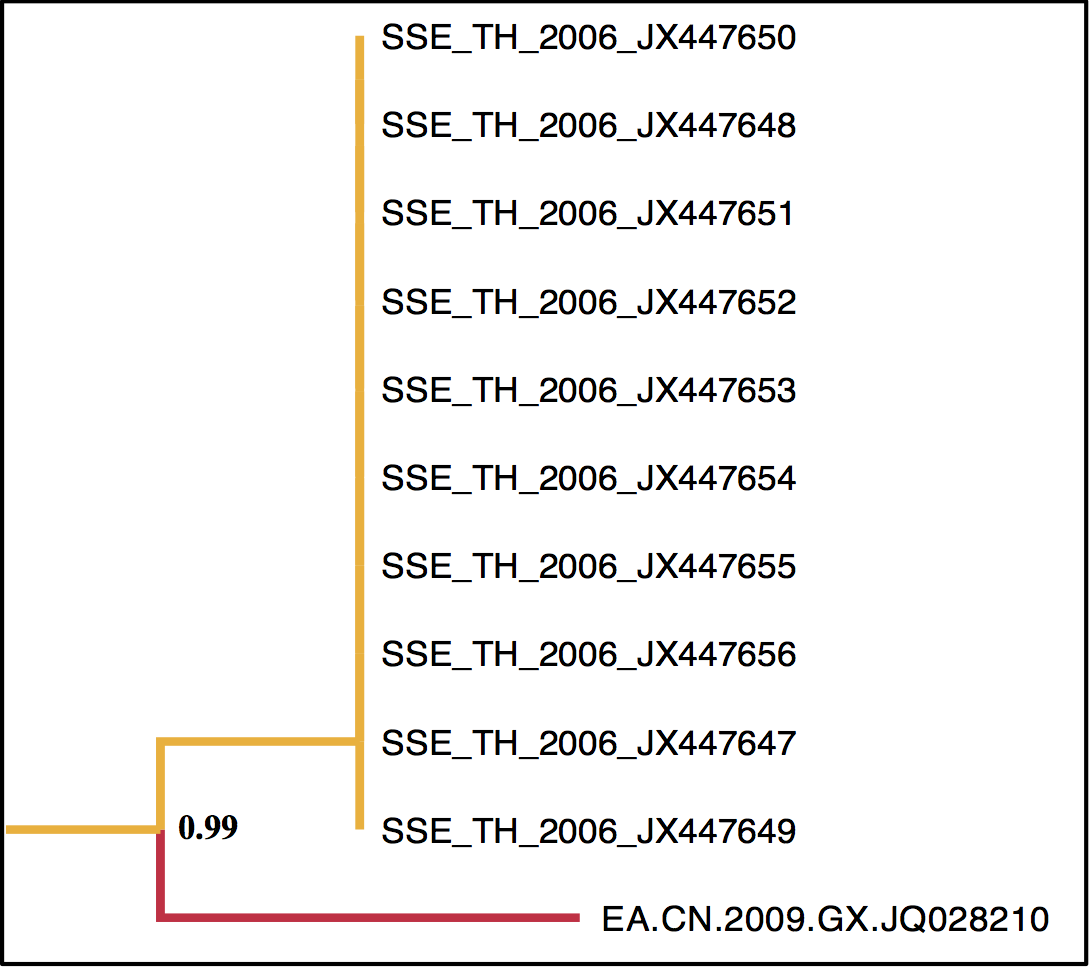


L2:


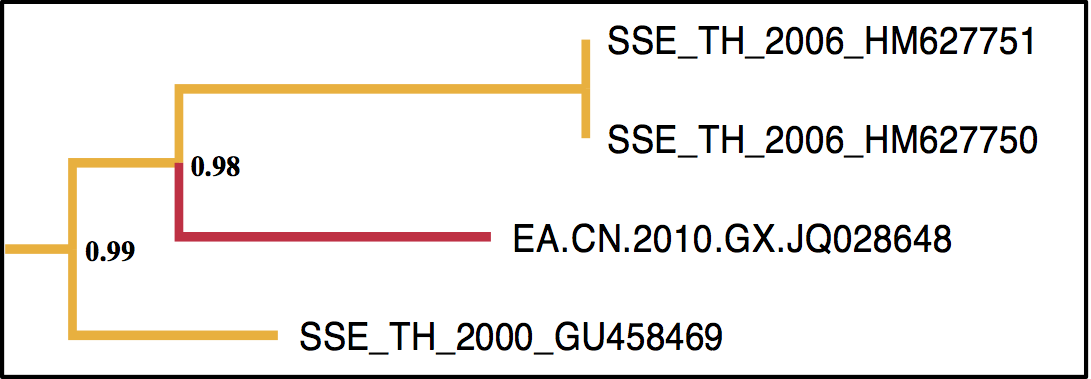


L3:


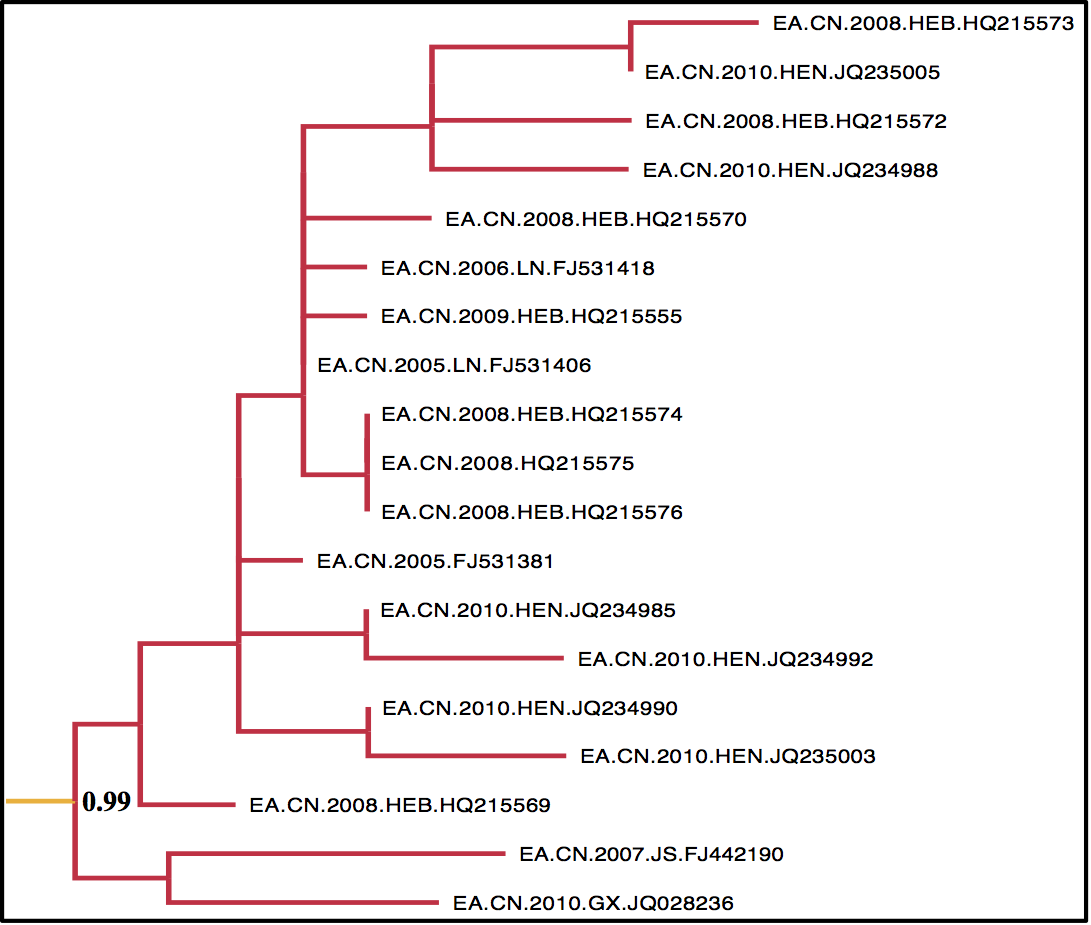


L4:


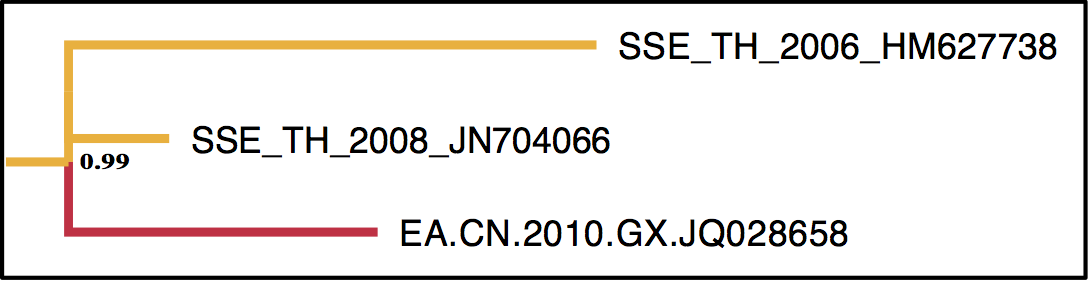


L5:


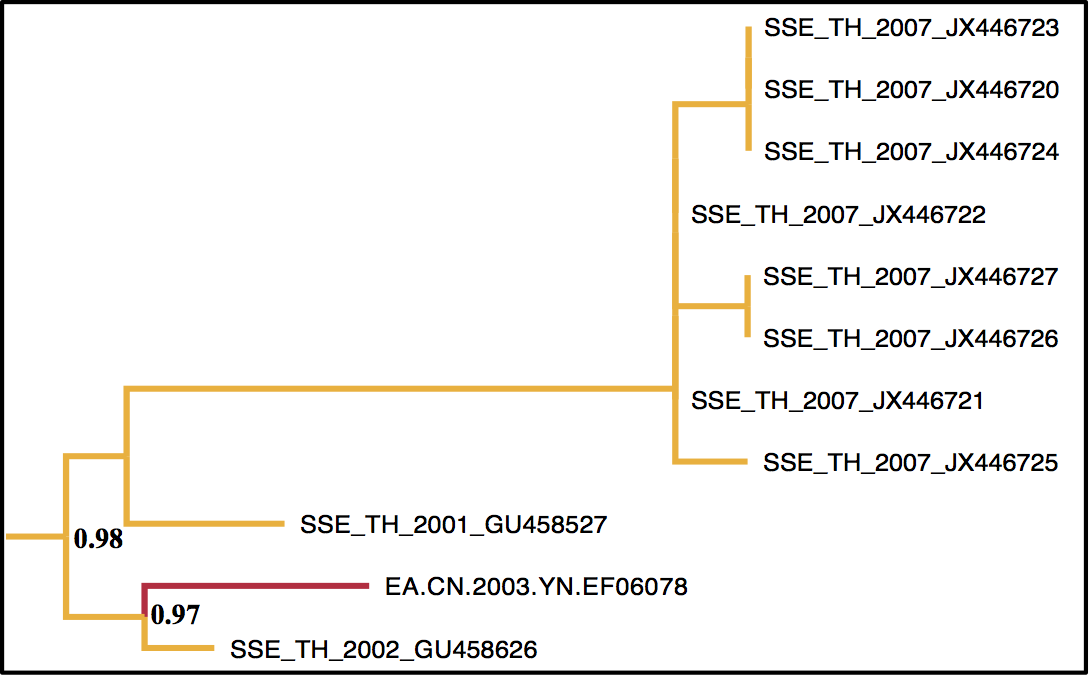


L6:


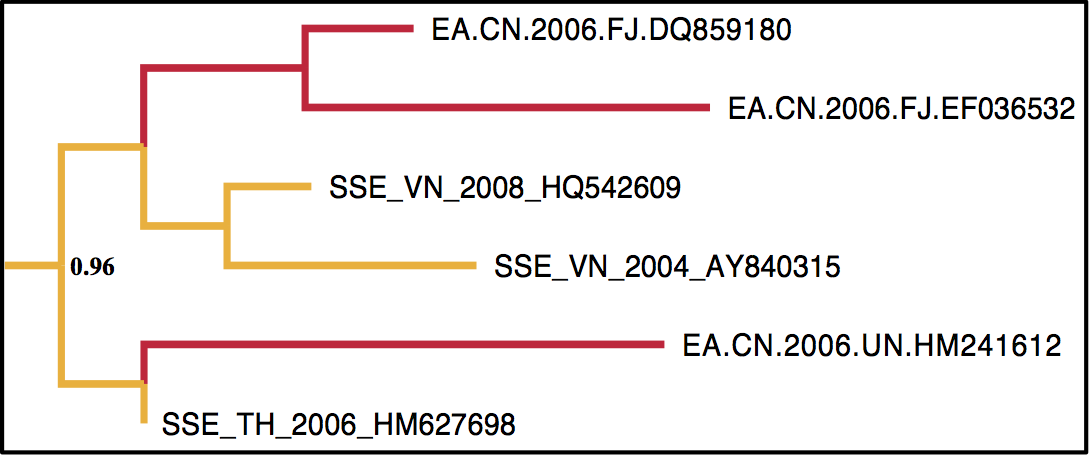


L7:


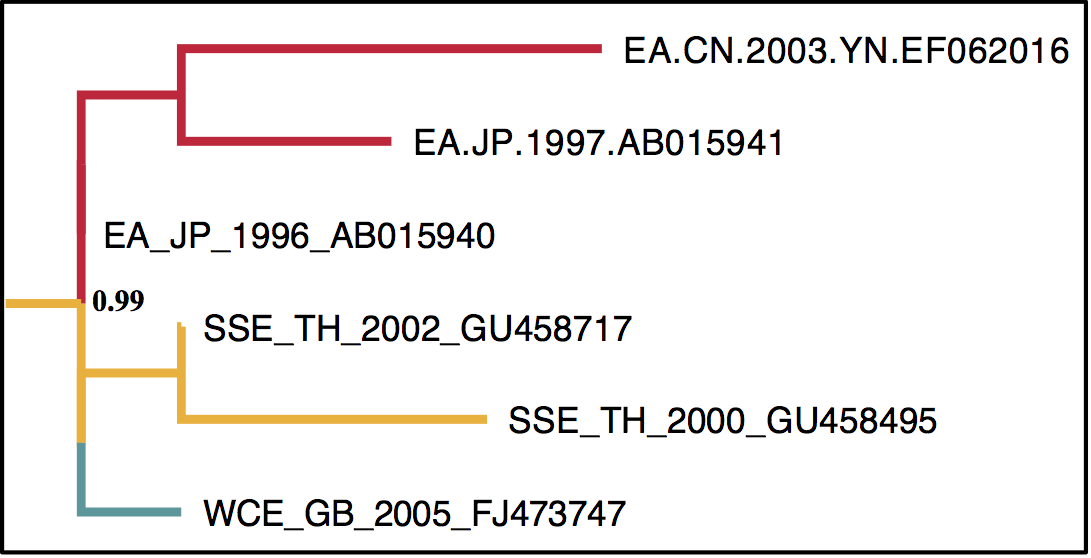


L8:


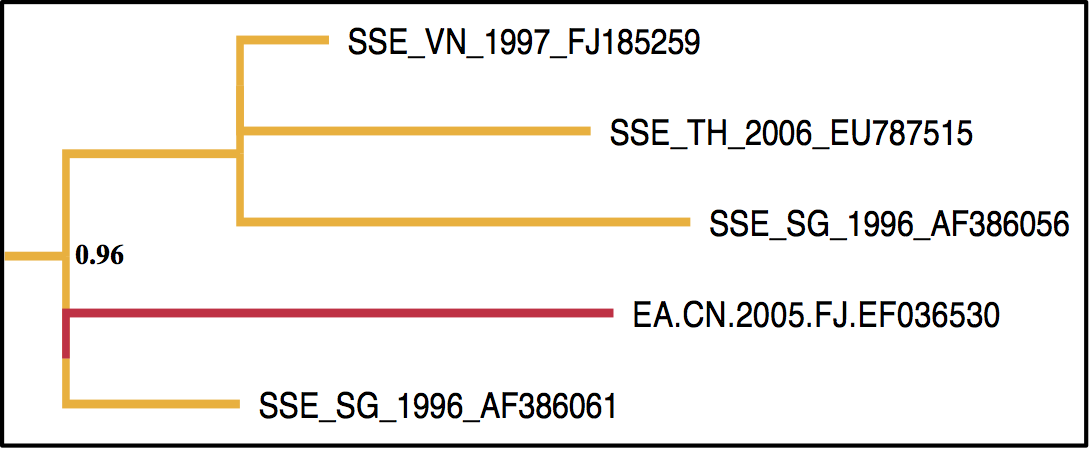


L9:


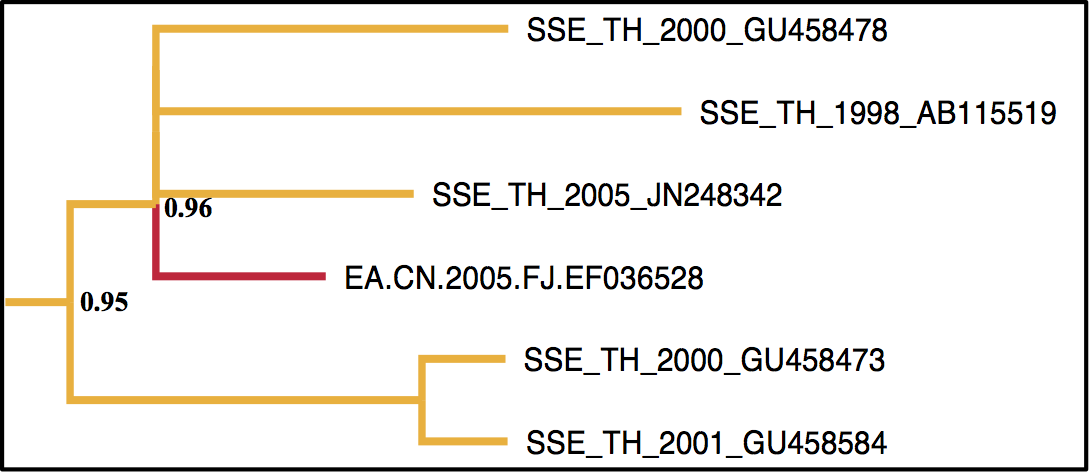


L10:


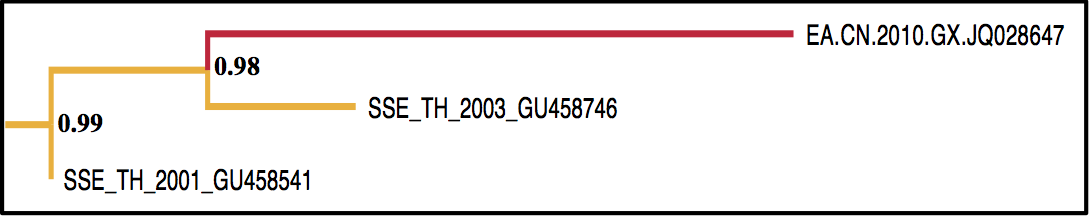


L11:


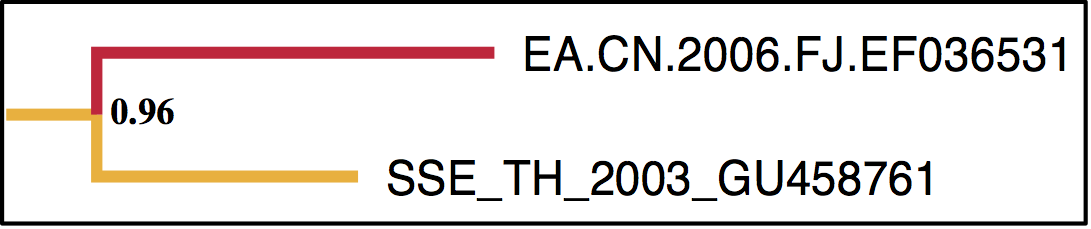


L12:


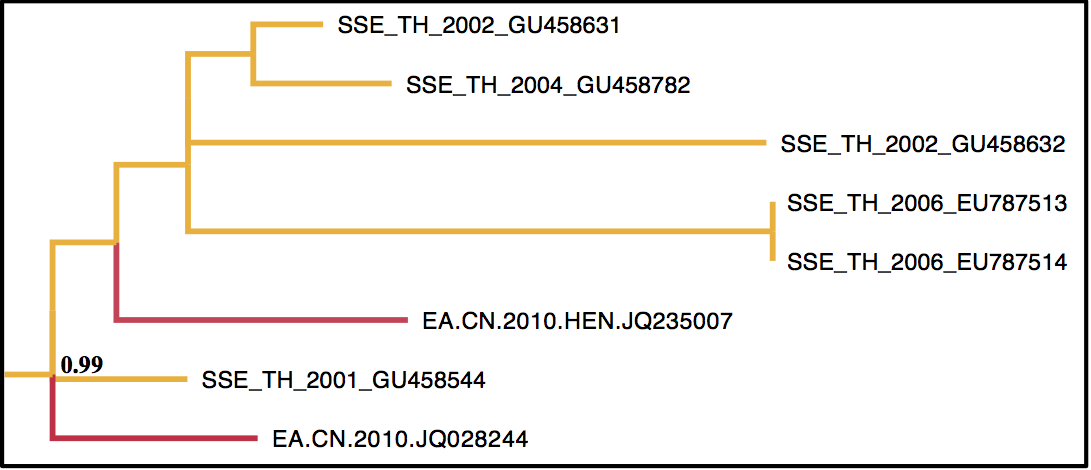


L13:


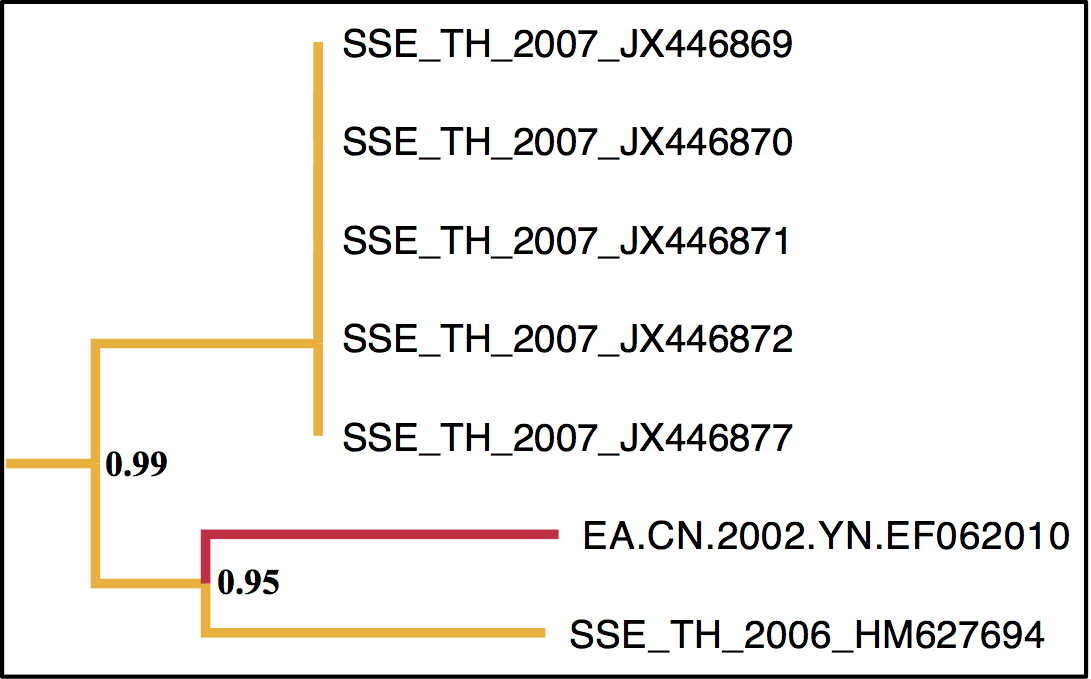


L14:


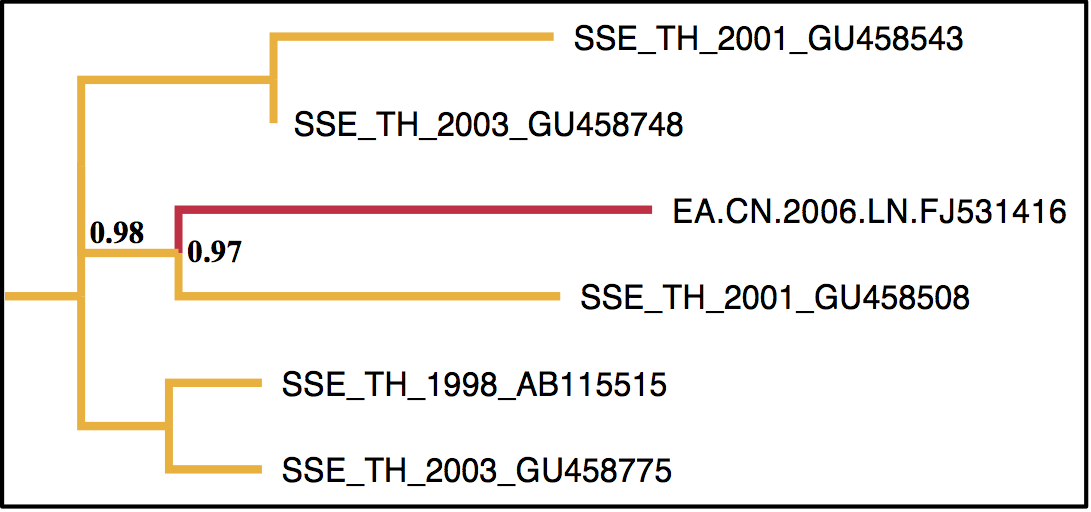


L15:


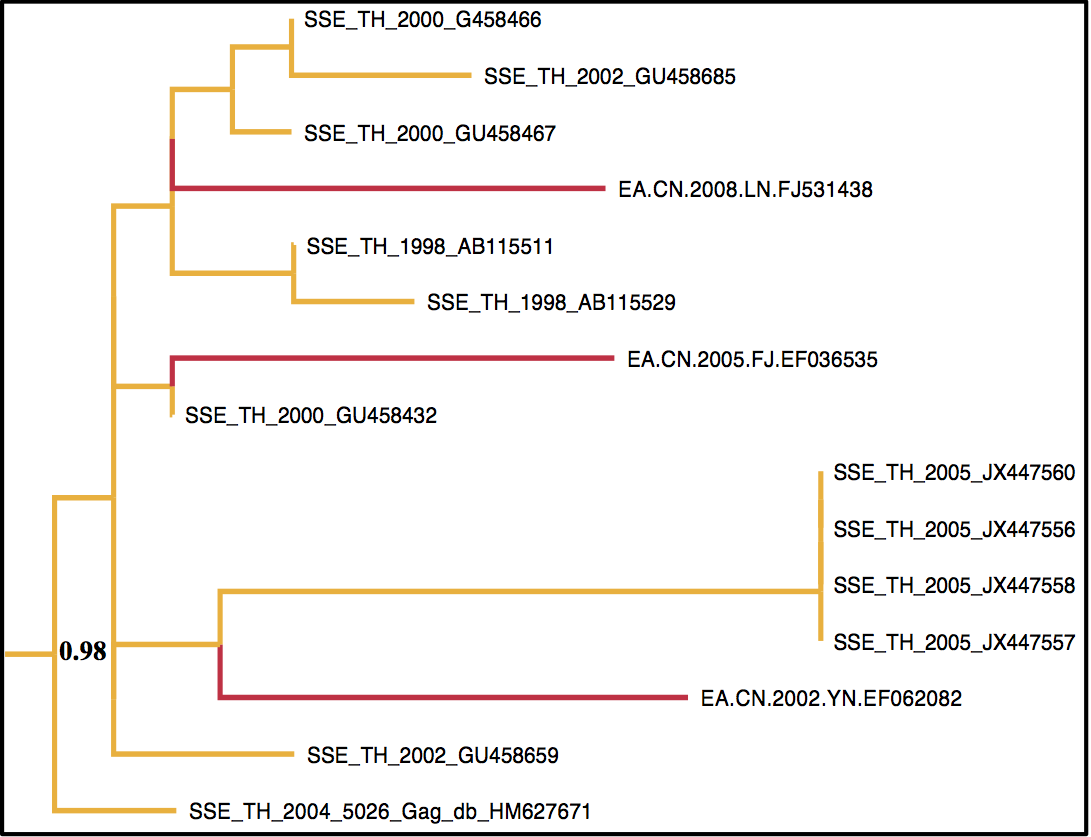


L16:


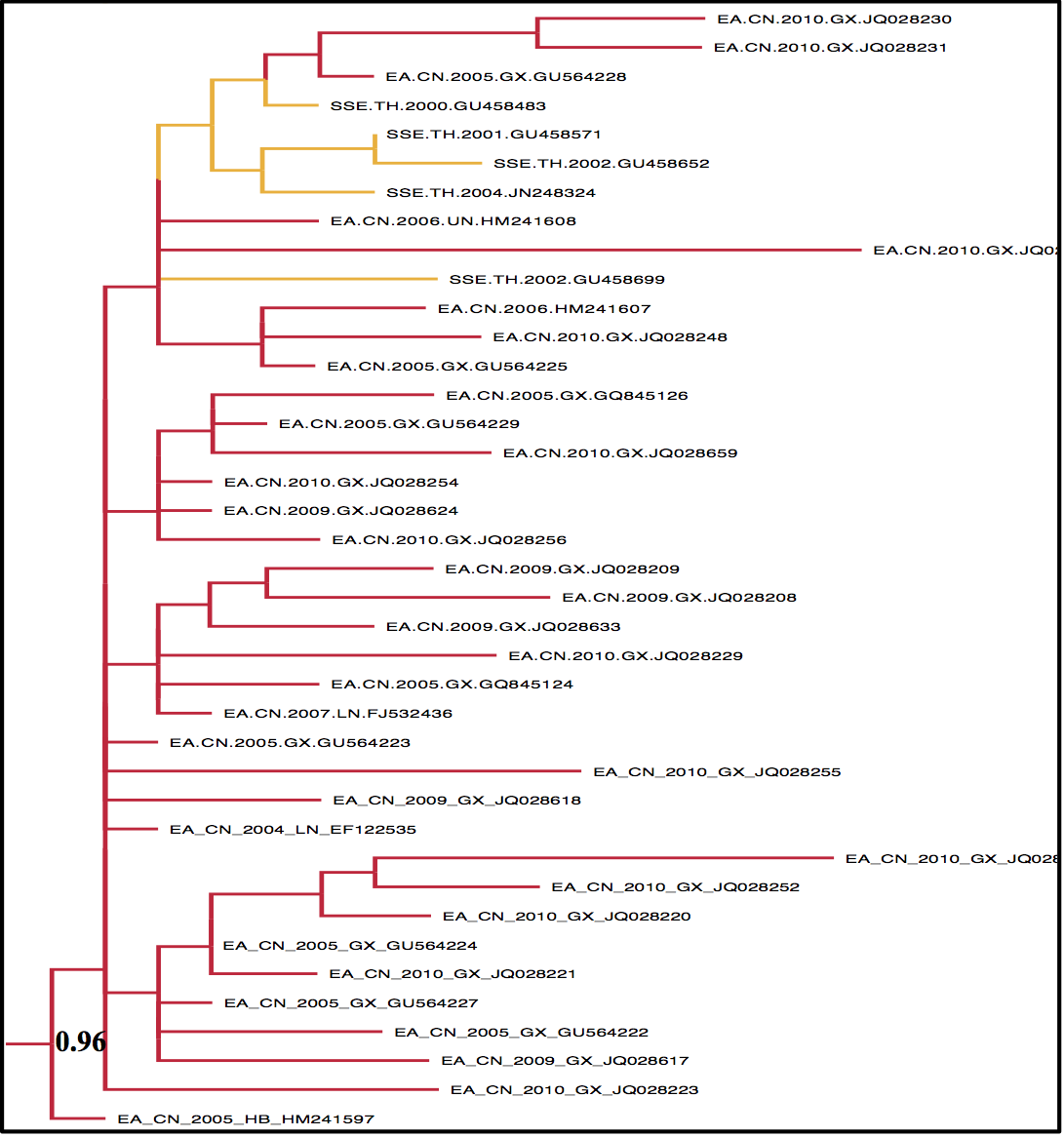


L17:


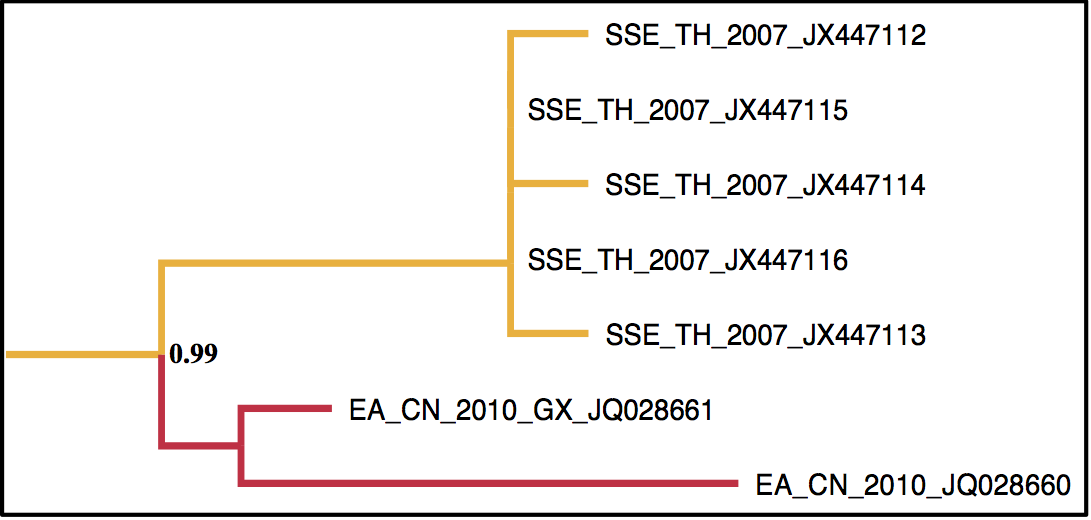


L18:


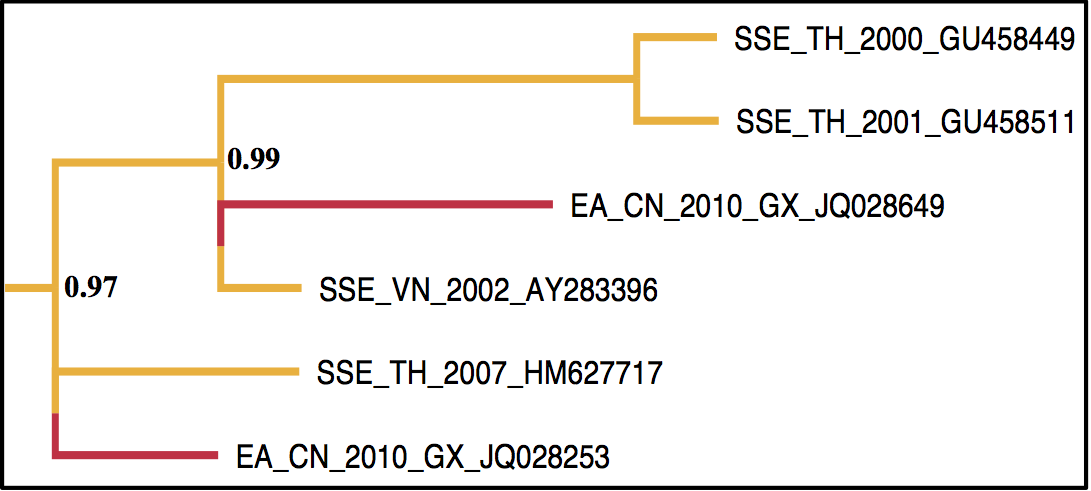


L19:


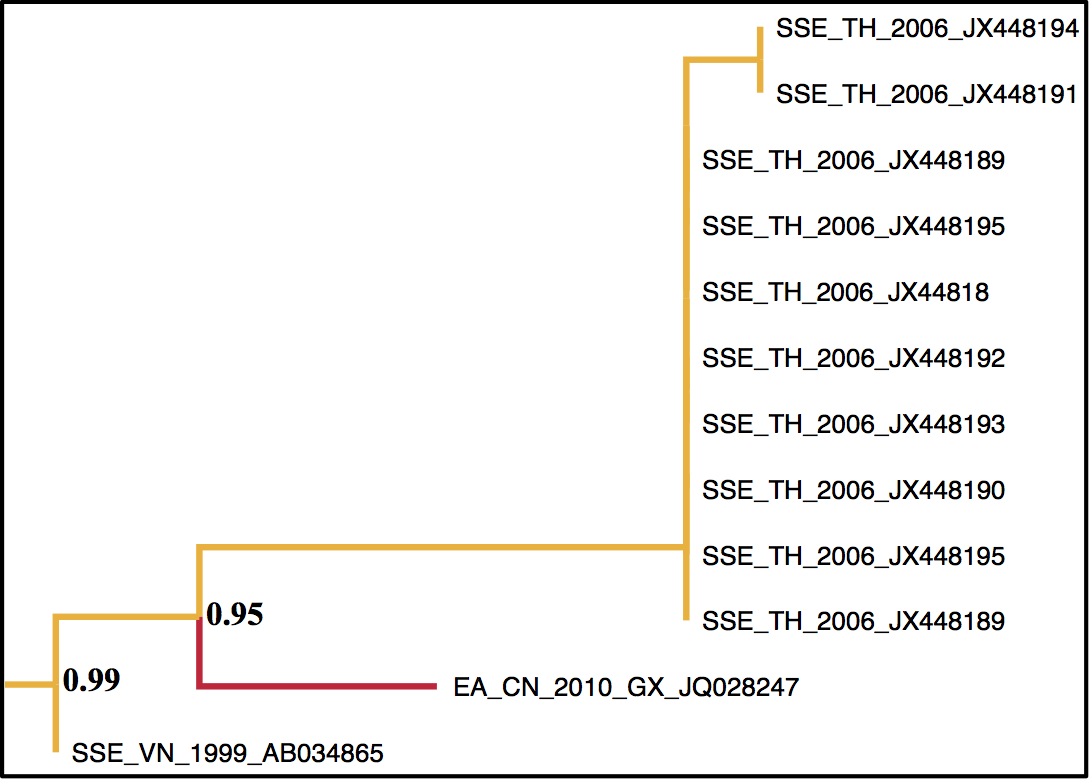


L20:


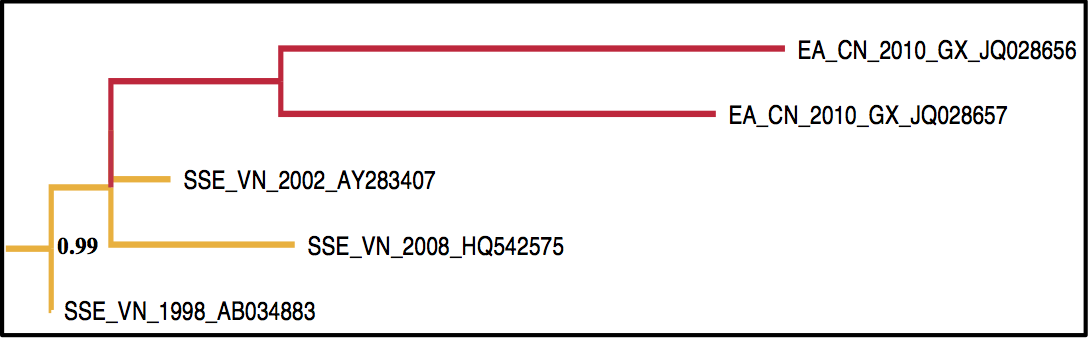


L21:


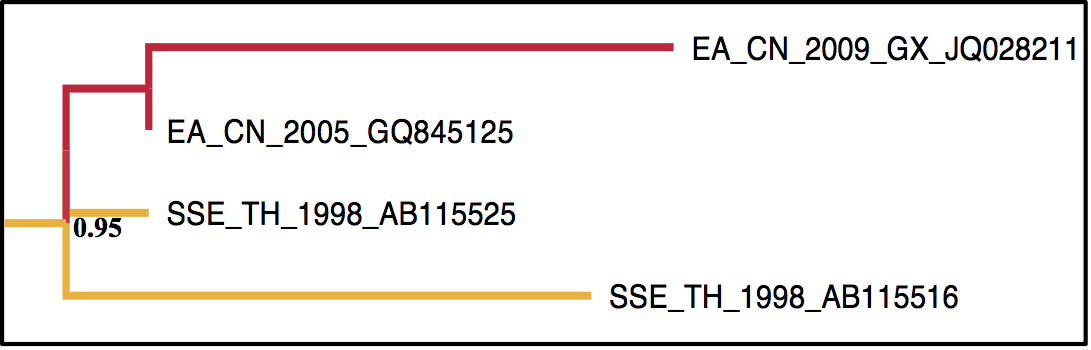


L22:


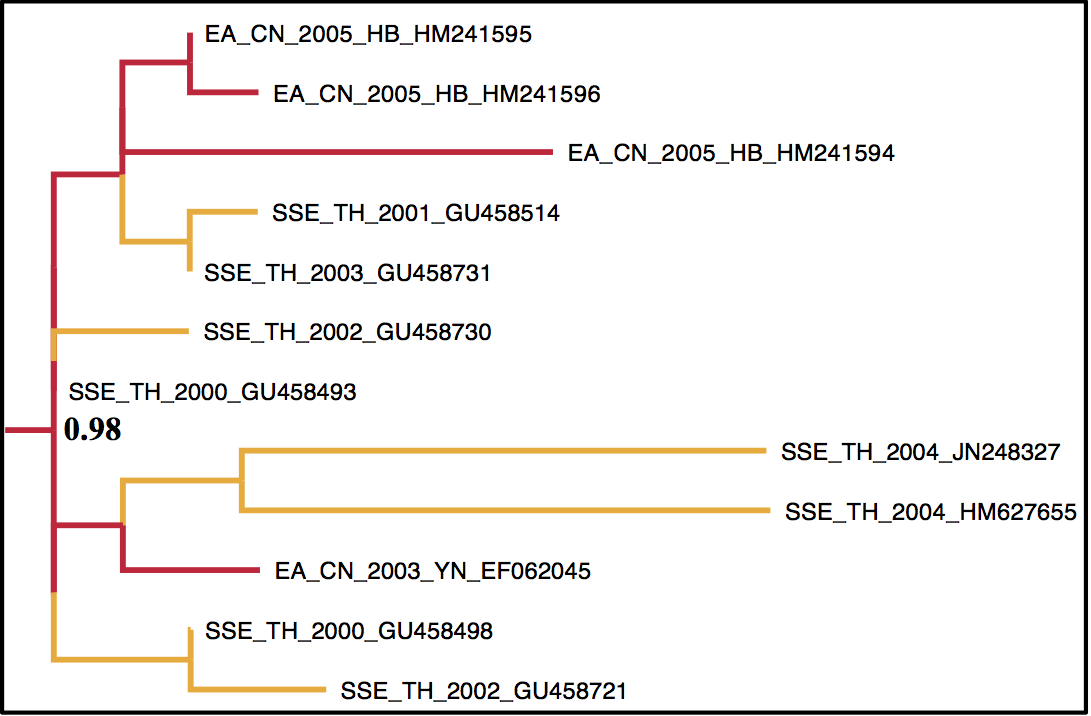


L23:


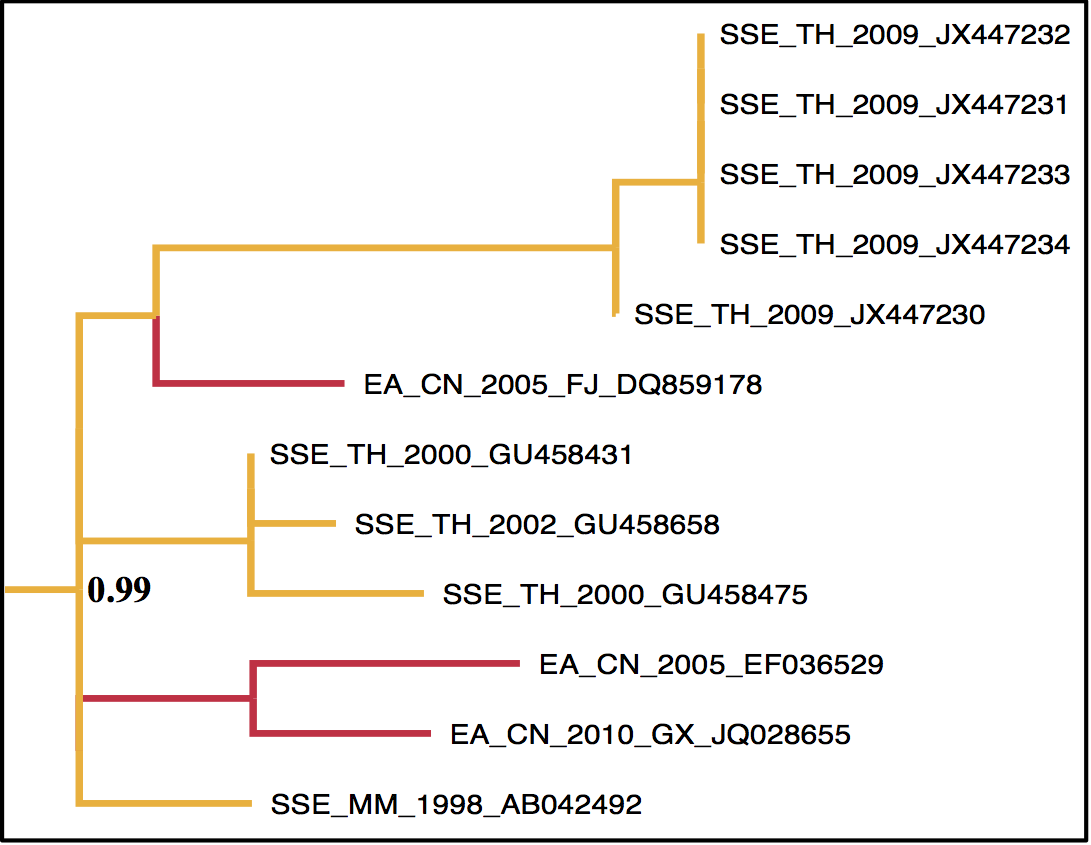


L24:


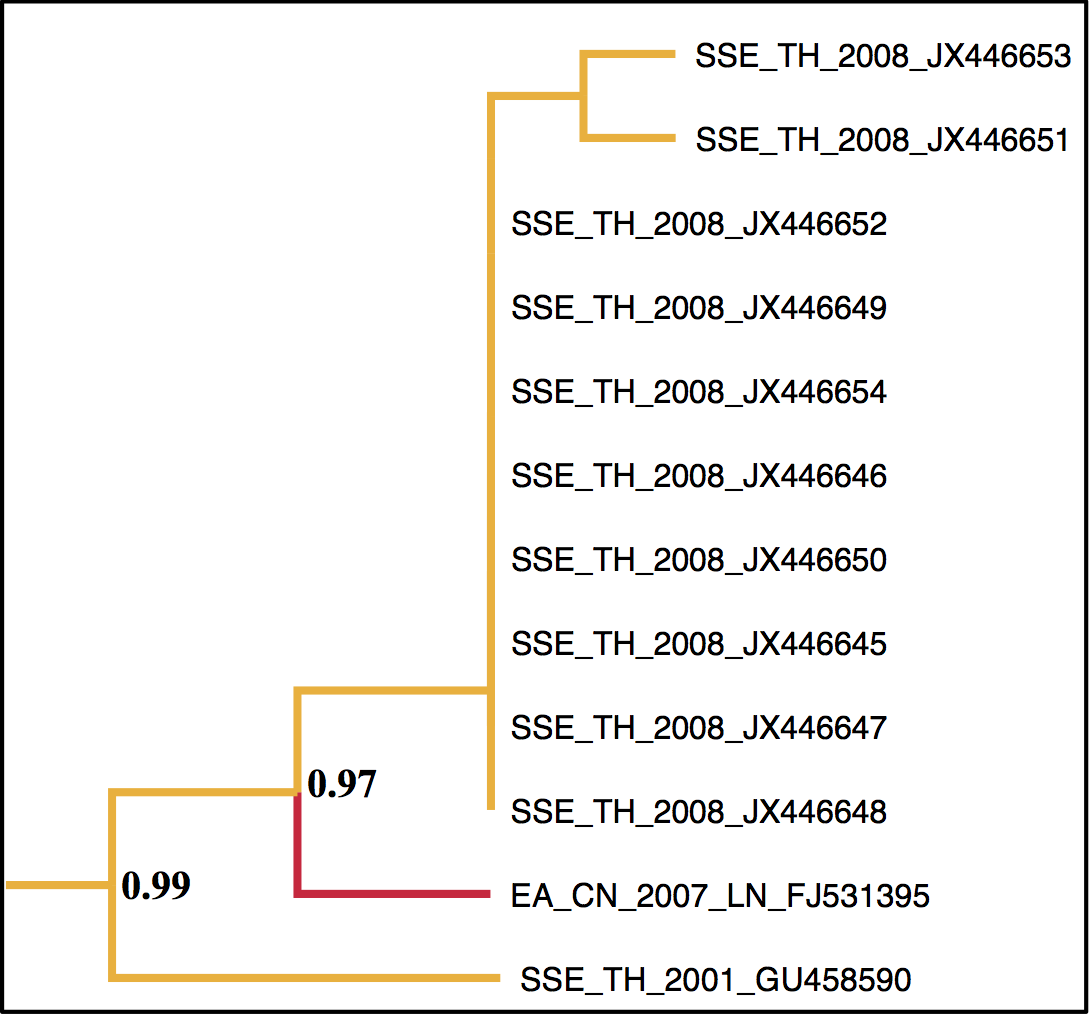


L25:


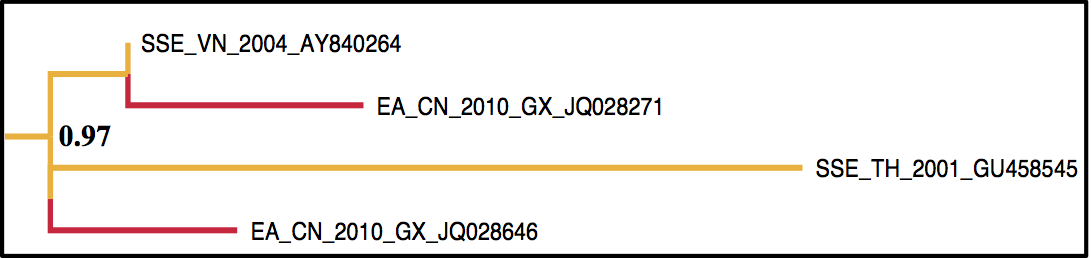


L26:


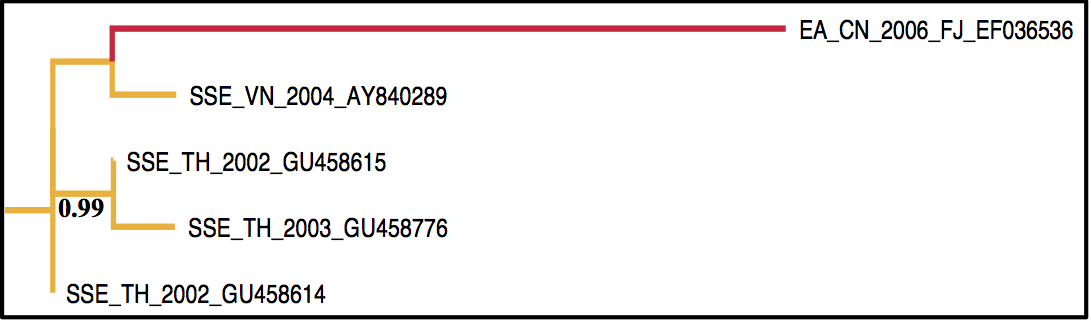


L27:


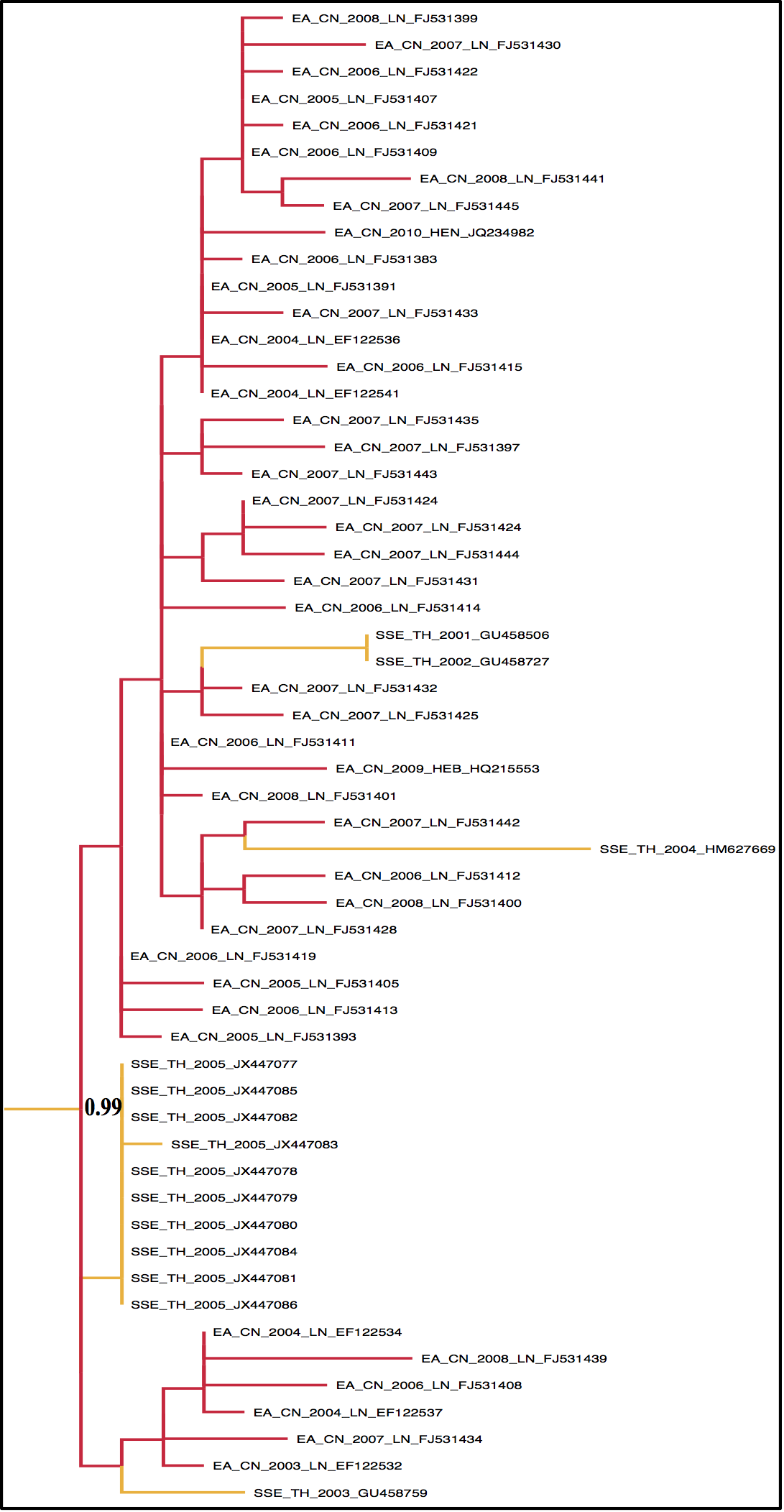

Supplement: Figure S1 — Close view of ML lineages containing Chinese CRF01_AE clades and the most closely related global sequences. The color of branches represents the geographic region from where the sequence originated, as explained in Figure 1. The names of CRF01_AE strains include reference to geographic region, country of origin, year of isolation, province (in case of Chinese sequences) and GenBank Accession number. Geographic regions represented are South and Southeast Asia (SSE), West and Central Europe (WCE), and East Asia (EA). Countries represented are Thailand (TH), Vietnam (VN), Japan (JP), Singapore (SG), Myanmar (MM), Great Britain (GB), and China (CN). Chinese provinces represented are Yunnan (YN), Guangxi (GX), Fujian (FJ), Liaoning (LN), Hebei (HEB), Hubei (HUB) and Henan (HEN). The aLRT support values are indicated only at key nodes. (DOCX) [file pone.0080487.s001.docx]
